# Secret Ingredients: Who Knows What's in Your Food?

**DOI:** 10.1289/ehp.121-a126

**Published:** 2013-04-01

**Authors:** Wendee Nicole

**Affiliations:** Wendee Nicole, based in Houston, TX, has written for *Nature*, *Scientific American*, *National Wildlife*, and other magazines.

British chef and food activist Jamie Oliver ignited a firestorm in January 2011 when he mentioned on the *Late Show with David Letterman* that castoreum, a substance used to augment some strawberry and vanilla flavorings, comes from what he described as “rendered beaver anal gland.”^1^ The next year, vegans were outraged to learn that Starbucks used cochineal extract, a color additive derived from insect shells, to dye their strawberry Frappuccino^^®^^ drinks^2^ (eventually, the company decided to transition to lycopene, a pigment found in tomatoes^3^).

Although substances like castoreum and cochineal extract may be long on the “yuck factor,”^4^ research has shown them to be perfectly safe for most people; strident opposition arose not from safety issues but from the ingredients’ origins. But these examples demonstrate that the public often lacks significant knowledge about the ingredients in foods and where they come from.

This is not a new development; the public relationship to food additives has a long history of trust lost, regained, and in some cases lost again. The Federal Food, Drug, and Cosmetic (FD&C) Act of 1938^5^ was passed shortly after the deaths of 100 people who took an untested new form of a popular drug, which contained what turned out to be a deadly additive.^6^ The new law was consumer oriented and intended to ensure that people knew what was in the products they bought, and that those products were safe.

The law has been amended over the years in attempts to streamline and bring order to the sprawling task of assessing and categorizing the thousands of substances used in foods, drugs, and cosmetics. One result of this streamlining is that under current U.S. law, companies can add certain types of ingredients to foods without premarket approval from the thin-stretched Food and Drug Administration (FDA). In other words, there are substances in the food supply that are unknown to the FDA. In 2010 the Government Accountability Office (GAO) concluded that a “growing number of substances … may effectively be excluded from federal oversight.”^7^ Is this a problem? The answer depends on whom you ask.

## A Brief History of Regulation

Foods contain ingredients intentionally added for a specific purpose, such as vitamins, preservatives, flavorings, and colorings (“direct additives”) and those that are added unintentionally through processing, storage, or packaging (“food-contact substances” or “indirect additives”).^8^ The modern era of their regulation in the United States began when Congress passed the 1958 Food Additives Amendment^9^ to the 1938 FD&C Act, which mandated premarket approval and established safety standards.

The 1958 amendment prohibits the FDA from considering an ingredient’s benefits during the approval process, only how safe it is. It also includes the Delaney Clause, which bans any ingredient that has been shown to cause cancer to animals or humans at any dose. Pesticide residues were originally included in the Delaney Clause but were removed with the 1996 Food Quality Protection Act.^10^ A separate amendment in 1960 mandated premarket approval for all color additives, both synthetic colors (e.g., FD&C Blue 2) and “natural” colors derived from animal, vegetable, and mineral sources (e.g., cochineal extract).^11^

The FDA regulates colors separately from other additives. The FDA currently certifies nine synthetic dyes—FD&C Blue 1, Blue 2, Green 3, Red 3, Red 40, Yellow 5, Yellow 6, Citrus Red 2 (used only on orange peels), and Orange B (which is no longer used because of safety concerns but is not banned).^12^ Although called “coal-tar dyes,” synthetic colors are now made from petroleum; the FDA certifies each batch to ensure it meets quality standards. The FDA also allows a number of natural colors, such as carmine, annatto, turmeric, beta-carotene, and caramel color, which do not receive agency certification. Companies must petition the FDA to create and use new color additives.

A company’s petition to use a new food additive must contain information on its intended effect, the foods it will be used in, and estimated consumption levels, as well as data on the substance’s safety, usually in the form of acute and long-term toxicity studies. From these data, the FDA develops an acceptable daily intake value, usually based on the dose found to cause no adverse effect in animals, multiplied by a safety factor.^13^

Even as the 1958 amendment created a strict safety standard for regulated additives, it exempted “prior-sanctioned” substances, which had already been approved for use in food, and substances that are “generally recognized as safe,” or GRAS.^14^ GRAS determinations are based on scientific data, expert knowledge, and a history of common and apparent safe use of the substance in food. The FDA defines “safe” as “a reasonable certainty in the minds of scientists that the substance is not harmful under its intended conditions of use.”^15^ A GRAS substance is considered safe for specific intended uses in food with no review by the FDA unless data emerge that suggest a need to reconsider its safety.^7^

“Percentage-wise, flavors make up the largest number of GRAS ingredients in food,” says Mitchell Cheeseman, former acting director of the FDA’s Office of Food Additive Safety. Companies add flavorings of natural or synthetic origin to restore a food’s inherent or expected flavor, or to enhance it, because flavors present in unprocessed foods often change during processing.

Food labels need not include a flavoring’s chemical name or a complete listing of all the flavors present, says Cheeseman. “Part [of the reason] is protecting the industry’s trade secret formulations and part is that the label would be substantially longer than it is for most foods.” The U.S. Department of Agriculture National Organic Program disallows artificial flavors in certified organic foods but allows nonorganic natural flavorings.^16^

Companies have the authority to make their own GRAS determinations using a panel of qualified experts to review safety data. Each company then has the option—but not the requirement—of notifying the FDA of its panel’s findings. Beginning in 1960, the Flavor and Extract Manufacturers Association (FEMA), a trade organization, established its own GRAS determination process, independent of the FDA. FEMA’s 120 member companies submit GRAS applications to the organization’s expert panel, which publishes its results in the trade journal *Food Technology*. FEMA has declared more than 2,600 flavoring substances GRAS.^7^

But a company can’t just declare any substance GRAS—“there needs to be a scientific process to determine risk,” says David Acheson, a food industry consultant who worked as the FDA’s associate commissioner of foods until 2009. According to the FDA, “the scientific data and information about the use of a substance must be widely known, and there must be a consensus among qualified experts that those data and information establish that the substance is safe under the conditions of its intended use.”^15^

## Refining the Process

The first major issue with the GRAS system arose in the late 1960s when research showed that cyclamate salts, a GRAS-listed sweetener, caused cancer in rodents. As a result, the FDA banned cyclamate salts, and President Richard Nixon ordered the FDA to review all GRAS substances; ultimately, 422 of the substances initially listed were reviewed.^7^

In the early 1970s, the FDA began a new GRAS affirmation process that was similar to the process for regulated additives but voluntary: The FDA announced new GRAS petitions in the *Federal Register*, sought public comment, reviewed all data received, and affirmed or denied the GRAS status.^7^ But in 1997, citing limited staff and resources, the FDA proposed a new voluntary notification program. Under the new process, rather than publishing GRAS notices and requesting comments, the FDA reviews notices internally, and instead of affirming or denying a determination, it sends one of three possible responses: a “no questions” letter, a letter stating “insufficient information exists to make a GRAS determination” (this could be due to safety concerns or a lack of data), or a notice that the FDA has ceased to evaluate the notice at the company’s request.

“No questions” letters mean just that: The FDA has no questions about the company’s assertion of safety; these letters explicitly state that the FDA “has not … made its own determination regarding the GRAS status.” According to the GAO, receiving such a letter “improves the company’s ability to market its GRAS substance” to prospective buyers.^7^

With a notice of insufficient information, the company may decide either not to use the ingredient or to conduct additional research and resubmit its GRAS notice. From 1998 through 2008, 274 notifications were submitted, more than three-quarters of which received a “no questions” letter.^7^

**Figure:**
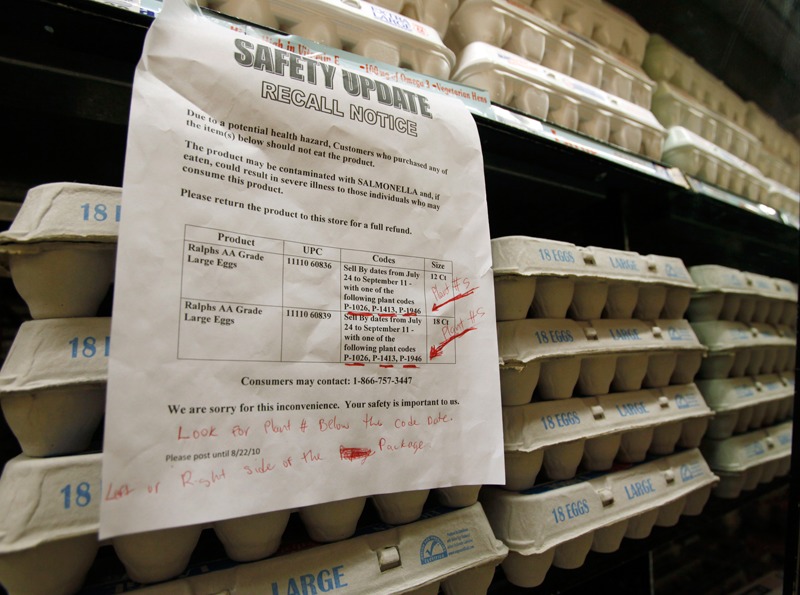
Generally speaking, consumer concern about additives has declined over the last three decades in the United States. In a 1997 poll 21% of supermarket shoppers quizzed considered food additives a serious health hazard, down from 36% in 1987. When asked what concerns they had over food safety, fewer than 1% volunteered additives, preservatives, or artificial colors, whereas 69% mentioned bacteria.^8^ © Reed Saxon / AP / Corbis

“You submit the data, FDA reviews it, and you get back ‘no questions.’ That’s supposed to be the carrot,” says Acheson; the stick is if companies use the substance and the FDA later determines it’s a problem. “It’s a fluffy rule, it’s a fluffy process,” Acheson says. “No one pays it much attention from a relative risk perspective, and there are tons of substances that get used that are GRAS that are perfectly safe.”

Although the FDA has been operating under this plan since it was proposed in 1997, these rules have never been finalized. By one estimate, more than 3,700 GRAS substances have been added to foods without FDA notification since 1958.^17^ Engineered nanomaterials (for instance, antimicrobial nanofilms for use in food packaging) are among the substances that could conceivably be declared GRAS without FDA notification—a potential concern, given the limited scientific knowledge of the toxicity of such materials.^7^^,^^18^

In 2010 the GAO documented this and other concerns in the report *Food Safety: FDA Should Strengthen Its Oversight of Food Ingredients Determined to Be Generally Recognized as Safe (GRAS)*. The GAO concluded that the FDA’s oversight process “does not help ensure the safety of all new GRAS determinations.”^7^ “[One thing] we took issue with is whether companies are maintaining proper documentation,” says Alfredo Gomez, a director on the GAO’s Natural Resources and Environment team. In addition, at the time the FDA began implementing the 1997 rule on voluntary GRAS notifications, the agency “intended to conduct random audits, but when we talked to them they said they had not conducted such audits.”

**Figure:**
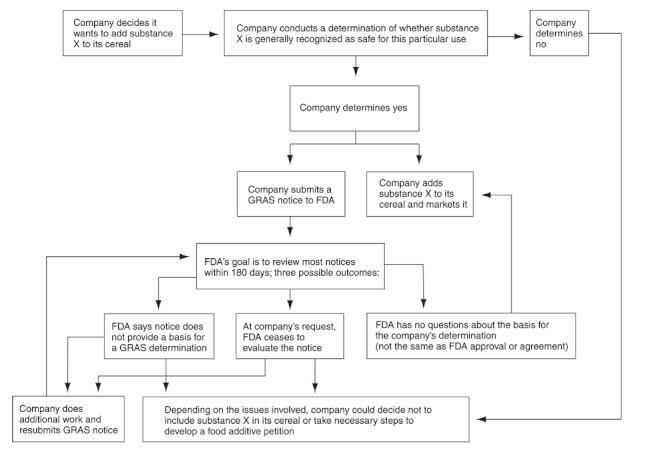
**Options available to a company participating in the voluntary GRAS notification program** This flowchart shows some of the potential courses of action a company might pursue under the FDA’s voluntary notification program; it does not show all possible variations. At any point in the process, a company might decide not to include the substance in its product or might proceed to market without a response from the FDA. Nevertheless, there must be evidence that the substance is safe under the conditions of its intended use. Reprinted from GAO (2010).^7^

The GAO report offered several recommendations, including requiring any company that conducts a GRAS determination to provide the FDA—and make publicly available—basic information about the determination. Other recommendations included spot-checking the appropriateness of company determinations, creating guidelines to minimize the potential for conflicts of interest on companies’ appointed expert panels, and finalizing the 1997 rule governing the voluntary notification program. The report further recommended the FDA use a more systematic method for reconsidering the safety of GRAS substances as new data emerge. And it called for a strategy to help ensure that engineered nanomaterials designated as GRAS without the agency’s knowledge are in fact safe.

“The agency has not yet fully implemented any of our recommendations,” Gomez says. However, in December 2010 the FDA opened a new public comment period on the 1997 rule with the intent of finalizing it. (As of the GAO’s last followup in May 2012, the agency was still reviewing these comments, according to Gomez.) And in April 2012 the FDA published draft guidance for manufacturers who want to use new nanoscale forms of existing food substances.^19^

## The Nature of Safety

In a 1995 article that gives insight into FEMA’s GRAS determination process, the group’s attorney and senior science advisor wrote, “Whether a food ingredient is GRAS depends on general recognition of safety, not on safety per se.”^20^ In other words, the authors asserted, even if a substance is toxic at higher doses, most substances are used at safe levels. The authors continued, “Recent advances in the understanding of tumor development have been noted by the FEMA Expert Panel and in several cases have enabled the Panel to conclude that a substance that causes tumors in laboratory animals at high doses is nevertheless GRAS under conditions of intended use in human food, because, for any of several reasons, the results from animal studies are not relevant to human safety.”

This interpretation is exemplified by the case of isoeugenol, which is extracted from cloves, basil, gardenias, and other plants and commonly added to drinks, chewing gum, and baked goods. The National Cancer Institute nominated isoeugenol for a National Toxicology Program (NTP) study because of its structural similarity to several carcinogenic compounds, including safrole, a sassafras-derived flavor banned in 1960. The NTP’s study, published in 2010, found “clear” evidence that isoeugenol caused liver cancer in male B6C3F_1_ mice and “equivocal” evidence of carcinogenicity in female B6C3F_1_ mice and male F344/N rats.^21^

Nevertheless, FEMA published a GRAS determination in *Food Technology* declaring isoeugenol safe. The paper called the tumors “a secondary biological response to dose-dependent hepatotoxicity” and pointed out that male B6C3F_1_ mice are predisposed to develop spontaneous liver tumors. “The occurrence of these neoplasms in the present study is considered a high-dose phenomenon without any relevance for assessing the potential cancer risk of the use of isoeugenol as a food flavor ingredient,” FEMA concluded.^22^

**Figure:**
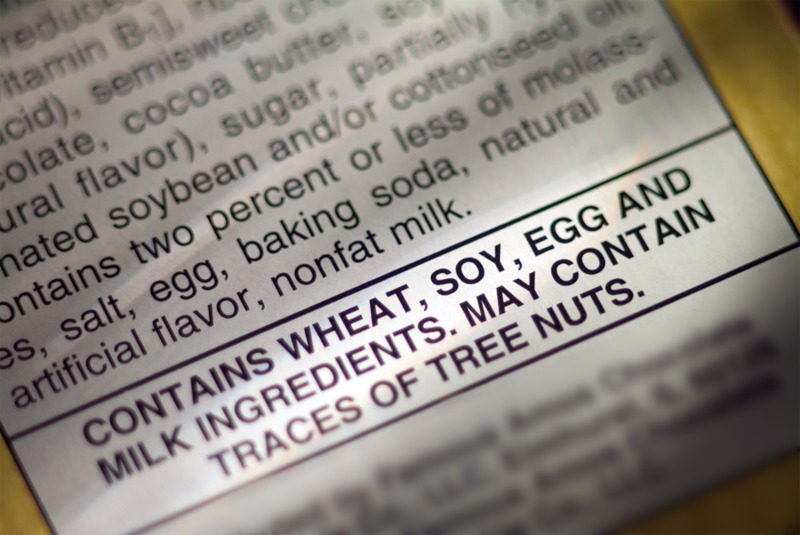
Food labeling can inform consumer choices. But people with limited means must often select the cheapest rather than the most nutritious option, and surveys indicate less-affluent people also are more likely to trust the safety of foods.^48^^,^^49^ © Envision / Corbis

The FEMA paper listed the estimated U.S. daily per-capita intake of isoeugenol flavoring at 0.02 μg/kg body weight (bw)/day, whereas the NTP tested concentrations of 75, 150, and 300 mg/kg bw/day. The World Health Organization has estimated isoeugenol intake in the United States at 40 μg/day.^23^

NTP’s Toxicology Branch chief Paul Foster, who worked on the study, says FEMA did not accurately represent the rodent study findings. “There was a clear increase of hepatocellular carcinoma in a dose-dependent manner,” says Foster. “‘Clear’ is the strongest level of evidence that we have when we draw conclusions about carcinogenic hazard. Something like eighty percent of male mice treated at the high dose level had liver tumors.”

FEMA “tried to dismiss the findings due to chronic liver toxicity,” Foster adds. “If their argument was valid, you would expect to see liver damage in animals that had not produced liver tumors, and you don’t. In the two-year study, animals without liver tumors did not show liver toxicity due to treatment with isoeugenol.” Furthermore, Foster says, “The whole point of undertaking cancer bioassays in rodents is because they can be predictive of human disease.” This was the basis behind Congressman James Delaney’s decision to include carcinogenicity not just in humans but also in animals in his controversial clause.

But the position that the Delaney Clause is far too restrictive to be implemented in practice is representative of the broader additive industry’s views.^10^ In 1981 the GAO, too, urged Congress to reevaluate the clause in light of technological advances and because of the inherent uncertainty of cancer risk assessment.^24^ Nevertheless, Congress has not changed the Delaney Clause, and subsequent court cases have upheld its application. A 1987 D.C. Circuit Court case (*Public Citizen v. Young*) upheld the clause for Orange nr 17, which has a lifetime cancer risk of 1 in 19 billion people; the judge stated that Congress intended an “extraordinarily rigid” position and that food manufacturers would find noncarcinogenic alternatives, and in fact, they did. The defendant appealed to the Supreme Court, which refused to hear the case.^25^

**Figure:**
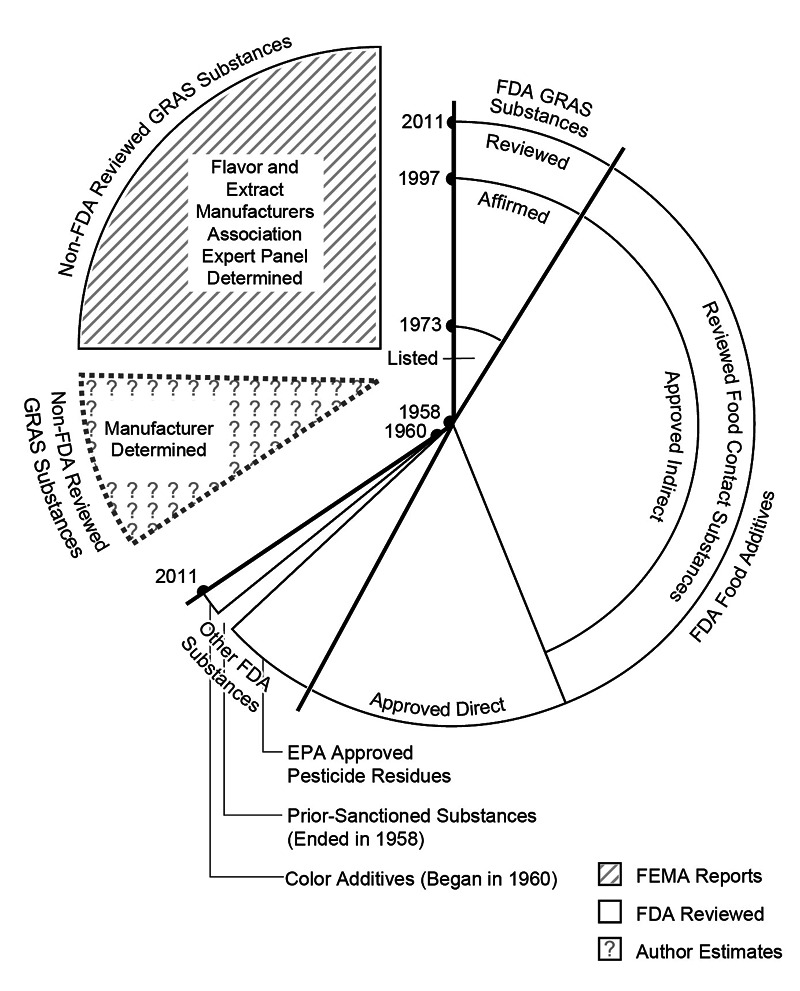
**Who determines safety?** This chart, reprinted with permission from Neltner et al. (2011),^17^ summarizes the contributions of the federal government, manufacturers, and various expert panels to affirmative safety decisions for the estimated 10,787 substances allowed in U.S. foods as of 11 January 2011. This number includes 4,646 GRAS substances, 5,292 food additives, and 849 “other” substances—color additives, pesticide chemicals/residues, and prior-sanctioned substances. Of the 4,646 GRAS substances, 2,702 were determined to be safe by FEMA’s Expert Panel, 1,000 were determined by independent manufacturers, and 944 were determined by the FDA. The federal government also made safety decisions on 1,483 direct food additives, 3,007 indirect food additives, 802 food-contact substances, 120 prior-sanctioned substances, 148 color additives, and 581 pesticide chemicals/residues. Most of these government decisions were made by the FDA; however, the U.S. Department of Agriculture approved the safety of some prior-sanctioned substances, and the U.S. Environmental Protection Agency approved the pesticide chemicals/residues. The years marked on the axis reflect the evolution of the FDA’s methods for reviewing and affirming safety. © 2011 The Pew Charitable Trusts

Events to date suggest citizen action may prove one of the most effective means of oversight: Anyone can petition the FDA with concerns about a GRAS substance, color additive, or food additive. The agency has received petitions for diacetyl (used as butter flavoring in microwave popcorn),^26^ monosodium glutamate,^27^ partially hydrogenated oils,^7^ and other additives.^7^ It was a 2001 petition submitted by the Center for Science in the Public Interest (CSPI) over undeclared allergens in foods^28^ that in 2011 resulted in the new requirement that cochineal extract be declared on labels rather than included under vague labeling such as “color added” or “artificial color.”^29^ It was at that point that consumers became aware of the nonvegan cochineal extract in Starbucks’ strawberry Frappuccino.

## Health Questions

In the late 1960s allergist Ben Feingold hypothesized that dyes and other additives may be linked to childhood behavior problems.^30^^,^^31^^,^^32^ Colors were singled out to test Feingold’s hypothesis, since there are far fewer of them, compared with flavorings and preservatives.^33^ Using “elimination diets” that removed all additives, investigators tested how colors alone or mixtures of additives affected childhood behavior, particularly symptoms of attention-deficit/hyperactivity disorder (ADHD). Over the years, research showed mixed results, and interest diminished but never disappeared.

Concern spiked again with the 2007 publication of results from a large, randomized, double-blind study in Southampton, United Kingdom, funded by the British Food Standards Agency.^34^ Researchers tested mixtures of several dyes plus the preservative sodium benzoate on children not previously diagnosed with ADHD. One of the mixtures—composed of sodium benzoate, Yellow 5, Yellow 6, and two dyes not used in the United States (carmoisine and Ponceau 4R)—was associated with significantly intensified hyperactivity, although a later analysis of the data by the European Food Safety Authority (EFSA) concluded the findings were not strong enough to be used as a basis for changing the standards for the relevant additives.^35^ EFSA did note that the inconsistency of the findings could indicate special sensitivity to food additives, or to colors in particular, among certain individuals.

Stateside, CSPI petitioned the FDA in 2008 to revoke approvals for all synthetic dyes except Citrus Red 2 and to require warning labels in the interim.^36^ In 2011 an FDA Food Advisory Committee heard expert comment and then voted by a four-fifths majority that the evidence did not support a causal relationship between consumption of synthetic color additives and adverse behavioral effects in children in the general population.^37^ By a much smaller majority (8 to 6), they decided against interim warning labels for products containing synthetic color additives. But the FDA has not closed the issue and is reportedly collecting data on current dye levels in foods with an eye toward revisiting estimated daily intake levels.

**Figure:**
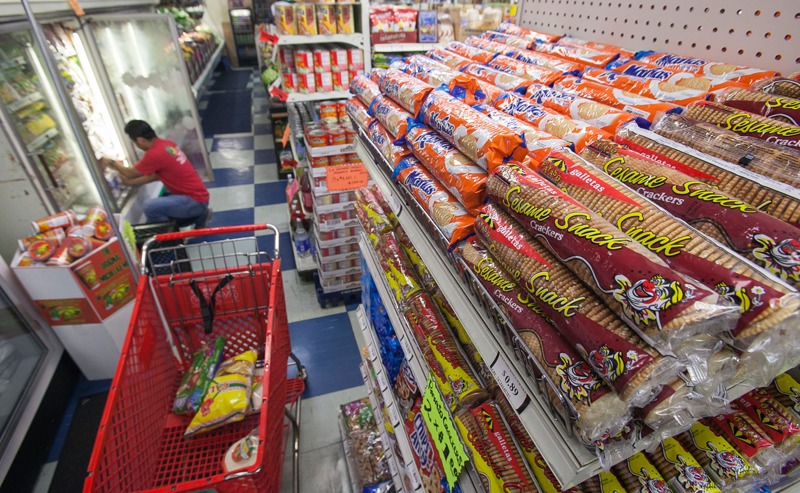
In the introductory chapter of an academic text on food additives, food scientist A. Larry Branen and agricultural expert Robert J. Haggerty wrote that although additives have allowed a great variety of foods with longer shelf lives, they also come with direct and indirect risks, not least of which is their contribution to “the increased availability of food products with a low density of nutrients.”^8^ The increased availability of junk food adds another layer of complexity atop safety concerns and regulation discrepancies, important in an era with obesity at an all-time high.^50^ © Damian Dovarganes / AP / Corbis

Taking a more precautionary approach, Britain and the European Union now require warning labels for foods with synthetic dyes and have encouraged food companies to transition to natural colors.^38^ According to Cheeseman, however, companies are moving away from synthetic dyes not because of safety concerns but because media coverage has convinced consumers there is a problem.

Other experts are convinced dyes do cause adverse health impacts in children, and possibly adults. Two separate meta-analyses compiled multiple studies to assess whether diets eliminating additives and colors were associated with reduction of ADHD symptoms.^39^^,^^40^ “We did see statistically significant effects,” says Joel Nigg, a psychiatry professor at Oregon Health & Science University and lead author of one of the meta-analyses. “That’s important because that in itself has not been believed until now.”

Although recent concern has focused on synthetic dyes, some naturally derived colors also may cause adverse health effects. For instance, there is evidence that cochineal extract may cause allergic reactions and asthma,^41^^,^^42^ and caramel coloring came under fire when it emerged that it can contain 4-methylimidazole (4-MEI), an animal carcinogen sometimes produced as a manufacturing by-product.^43^ In 2011 4-MEI was added to the list of carcinogens and reproductive toxicants maintained under California’s Proposition 65 (the Safe Drinking Water and Toxic Enforcement Act of 1986),^44^ and products containing more than 29 μg 4-MEI per serving sold in the state after January 2012 must carry a warning. To avoid the labels, PepsiCo and Coca-Cola now use a caramel color with low levels of 4-MEI in California.^45^

CSPI has petitioned the FDA to require labels for high-MEI caramel coloring nationwide.^46^ Meanwhile, colas from eight other countries tested by CSPI were found to contain 2–10 times the California limit of 4-MEI.^43^ A press release on the findings noted that people in other countries often drink much less soda than Americans do, so their exposure to 4-MEI is likely to be proportionately lower. “But now that we know it’s possible to almost totally eliminate this carcinogen from colas,” CSPI executive director Michael F. Jacobson was quoted as saying, “there’s no excuse for … companies not to do so worldwide, and not just in California.”^47^
