# Erratum: “Secret Ingredients: Who Knows What’s in Your Food?”

**DOI:** 10.1289/ehp.121-a180

**Published:** 2013-06-01

The April 2013 News article “Secret Ingredients: Who Knows What’s in Your Food?” [Environ Health Perspect 121:A126–A133 (2013] included a simplified version of Figure 1B from Neltner et al. [Navigating the U.S. food additive regulatory program. Compr Rev Food Sci Food Saf 10(6):342–368 (2011)]. In simplifying the figure, *EHP* introduced errors. We furthermore later determined that Figure 2 from Neltner et al. was more germane to the discussion than Figure 1B. Below is the appropriate figure in its unmodified original form with an updated caption.

**Figure:**
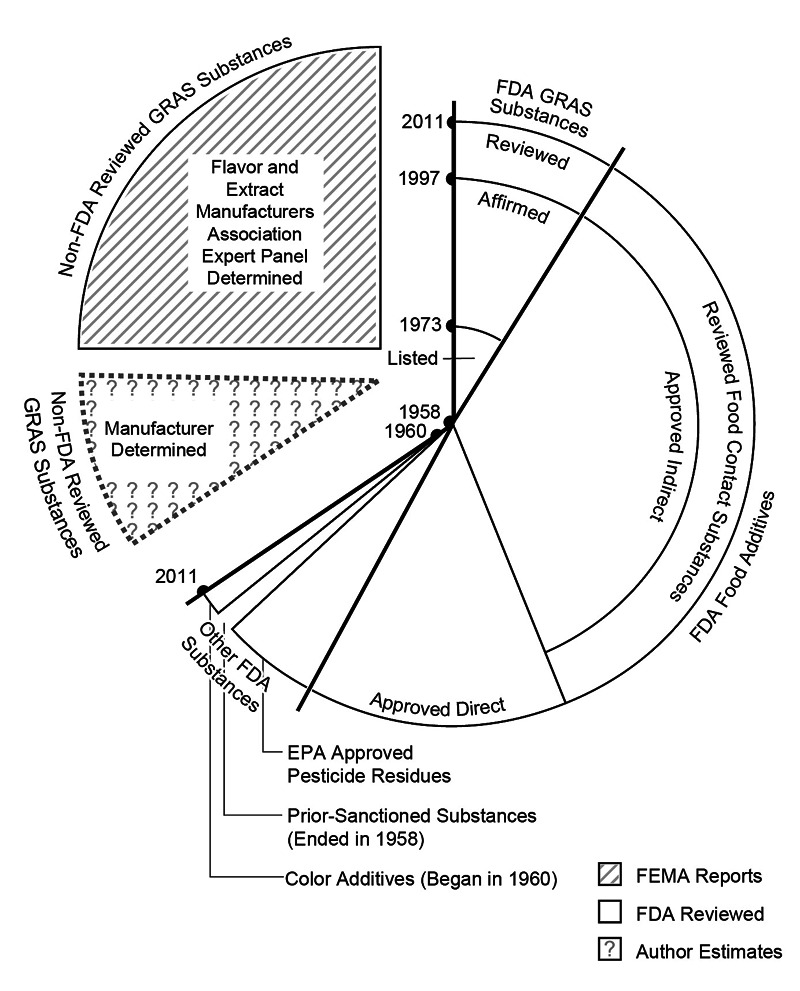
**Who determines safety?** This chart, reprinted with permission from Neltner et al. (2011),^17^ summarizes the contributions of the federal government, manufacturers, and various expert panels to affirmative safety decisions for the estimated 10,787 substances allowed in U.S. foods as of 11 January 2011. This number includes 4,646 GRAS substances, 5,292 food additives, and 849 “other” substances—color additives, pesticide chemicals/residues, and prior-sanctioned substances. Of the 4,646 GRAS substances, 2,702 were determined to be safe by FEMA’s Expert Panel, 1,000 were determined by independent manufacturers, and 944 were determined by the FDA. The federal government also made safety decisions on 1,483 direct food additives, 3,007 indirect food additives, 802 food-contact substances, 120 prior-sanctioned substances, 148 color additives, and 581 pesticide chemicals/residues. Most of these government decisions were made by the FDA; however, the U.S. Department of Agriculture approved the safety of some prior-sanctioned substances, and the U.S. Environmental Protection Agency approved the pesticide chemicals/residues. The years marked on the axis reflect the evolution of the FDA’s methods for reviewing and affirming safety. © 2011 The Pew Charitable Trusts

*EHP* regrets the error.

